# The gender wage gap among health care workers across educational and occupational groups

**DOI:** 10.1093/haschl/qxad090

**Published:** 2023-12-27

**Authors:** Janette S Dill, Bianca K Frogner

**Affiliations:** Division of Health Policy and Management, School of Public Health, University of Minnesota, Minneapolis, MN 55455, United States; Center for Health Workforce Studies, Department of Family Medicine, School of Medicine, University of Washington, Seattle, WA 98195, United States

**Keywords:** gender inequity, gender wage gap, health care occupations, health care workforce

## Abstract

Women perform 77% of health care jobs in the United States, but gender inequity within the health care sector harms women's compensation and advancement in health care jobs. Using data from 2003 to 2021 of the Annual Social and Economic Supplement of the Current Population Survey (CPS), we measured women's representation and the gender wage gap in health care jobs by educational level and occupational category. We found, descriptively, that women's representation in health care occupations has increased over time in occupations that require a master's or doctoral/professional degree (eg, physicians, therapists), while men's representation has increased slightly in nursing occupations (eg, registered nurses, LPNs/LVNs, aides, and assistants). The adjusted wage gap between women and men is the largest among workers in high-education health care (eg, physicians, advanced practitioners) but has decreased substantially over the last 20 years, while, descriptively, the gender wage gap has stagnated or grown larger in some lower education occupations. Our policy recommendations include gender equity reviews within health care organizations, prioritizing women managers, and realigning Medicare and Medicaid reimbursement policies to promote greater gender equity within and across health care occupations.

## Introduction

The health care sector is an industry that is dominated by women,^1^ but gender inequity in pay exists within the health care sector, potentially contributing to lower lifetime earnings for women and deterring women to advance in health care occupations.^2,3^ However, little is known about how the gender wage gap has changed in the health care workforce over the last decade, especially in relation to changes in the gender composition of the health care workforce. Further, less is known about the gender wage gap at different levels of education, as most research has focused on physicians and nurses, with little examination of patterns across health care occupations. In this paper, we examine how the gender composition and the gender wage gap have changed over the last 2 decades across the full spectrum of the health care workforce.

### The gender wage gap and education

In the general workforce, women's annual earnings were 82.3% of men's in 2020,^4^ and this gender gap in pay has remained relatively stable in the United States over the past 15 years.^5^ For women of color, the gap is even wider; Hispanic women earned just 57 cents and Black women earned 64 cents for every $1 earned by White, non-Hispanic men in 2020.^6^ The wage gap between men and women exists at every level of educational attainment, despite women graduating at higher percentages than men. Today, 36% of young women have a bachelor's or a graduate degree, compared with 28% of young men.^7^ However, while higher levels of education lead to higher wages in general, more education for women does not necessarily lead to the same wage gains as it does for men.^8,9^ White women with a college degree earn approximately 74% of their college-educated male peers, while Black and Latina women with a bachelor's degree have the largest gap, at 65%, compared with White, non-Hispanic male peers.^4^ In other words, education has not effectively reduced the gender wage gap across the economy, even though women are now substantially more educated than men.

### The gender wage gap across health care occupations

Within the health care workforce, gender wage gaps in the physician workforce are well established. Male physicians earn higher wages than women physicians across all medical specialties, and medical specialties that are dominated by men pay significantly more as compared with specialties where women are concentrated.^10-12^ A recent estimate is that female physicians earn approximately 25% less than male physicians (>$2 million over their career),^3^ and the gap has persisted over decades.^12^ Among a relatively homogenous group of physician researchers, male gender was associated with higher salary, even after adjustment for specialty, academic rank, leadership positions, publications, and research time.^13^

The gender wage gap persists among other health care workers with high levels of training and education, including nurse practitioners.^14,15^ A study that looked comprehensively across health care professionals found a gender earnings gap for pharmacists, dentists, physician assistants, and health care executives, and the wage gap fell consistently only for health care executives and pharmacists over time.^16^ Among nurses, male RNs out-earn female RNs across settings, specialties, and positions, with no narrowing of the pay gap over time.^17,18^ Finally, few studies have examined the gender wage gap among health care workers with an associate’s degree or less, which represent a large share of health care occupations and are dominated by women. Past research has shown that, among among direct care workers, there is a persistent gender wage gap,^19-21^ even though very few men hold direct care jobs.

### Explaining the gender wage gap

What accounts for the gender wage gap? Across occupations, occupational segregation—where men and women work in specific occupations—accounts for almost half of the overall gender wage gap.^8,22,23^ Women-dominated occupations pay less, often much less, than male-dominated occupations, when controlling for education, skill level, work experience, and other factors.^22,24^ Folbre et al^25^ argue that women-dominated care sector industries like health care are less able then providers of business services to capture value added or extract rents because limited consumer sovereignty, incomplete information regarding quality, and large positive externalities reduce their relative market power. They argue that women's concentration in care services like health care explains a significant proportion of the gender wage gap.^25^

Within health care occupations, one explanation of the gender wage gap is the “glass escalator.” Many health care occupations—including nursing and direct care occupations like nursing assistants and home health aides—are heavily dominated by women. Research has shown that men in women-dominated occupations have higher wages and are more likely to be promoted compared with their women peers.^26,27^ These patterns likely reflect some preference by employers for masculine traits and a willingness to reward men at a higher rate than women.^28,29^ The glass escalator also likely reflects some selection effects, either by the individual or imposed by society; past research has shown that, within nursing, men are more likely to move laterally into specialties where there are higher wages, as well as more men, as compared with women.^27^

Additional factors that contribute to the gender wage gap include family implications for wages, including the “motherhood penalty” and the “fatherhood bonus,” as well as discrimination.^22,30-33^ For professional women, there is also evidence that women are excluded from the highest wage jobs because of the expectation of long hours and may opt for part-time work.^34^ During the pandemic, in which childcare became unstable, the percentage of women physicians with children significantly declined compared with men physicians with children, suggesting disproportionate responsibilities that women face in child rearing.^35^

### Importance of looking at the gender wage gap over time

Some health care occupations have seen a growing representation of women (eg, physicians), but some occupations, such as nursing, have seen a growing entry of men and decline in women's representation.^17,36^ In this study, we examined the wage gap between men and women comprehensively across health care jobs over time, and how the gap may be related to the representation of women in the field. To guide our examination, we used the “glass escalator” theory, which suggests that men will experience higher wages and career advancement than their women counterparts, and a greater presence of men will widen the gender wage gap. We hypothesized a negative association between the gender representation (eg, lower representation of women) and the size of the gender wage gap (eg, higher wage gap) across education and health care occupational categories. The results from this study will help policymakers and employers identify opportunities to address wage disparities.

## Data and methods

### Sample

This study is a retrospective analysis of cross-sectional observational data across multiple years. We used data from 2003 to 2021 of the Annual Social and Economic Supplement (ASEC; also known as the March Supplement) of the Current Population Survey (CPS), a public data set jointly collected by the Bureau of Labor Statistics and US Census Bureau and made available via the IPUMS-CPS.^37^ The CPS is a nationally representative monthly survey of approximately 60 000 households. Individual-level survey weights provided in the CPS for generalizability were used for all analyses. The ASEC provides detailed data on wages and other income, job characteristics, and sociodemographic characteristics, including information on an individual's occupation and industry in the current year and in reflection to the prior year.

We divided survey respondents into those self-reporting as woman vs man. Our sample includes individuals employed in a health care occupation as defined by US Census occupation codes in the current year of being surveyed. We categorized health care workers based on the highest level of education they have achieved: less than a high school degree, high school degree or GED (General Educational Development) equivalent, postsecondary education, associate’s degree, bachelor's degree, master's degree, or doctoral/professional degree. Following groupings used in studies using CPS data and allowing for comparability, we also categorized the over 50 health care occupations into 1 of 9 occupation groups: physicians, advanced practitioners (eg, pharmacists, dentists, physician assistants/associates), advanced practice registered nurses, registered nurses (RNs), licensed practical/vocational nurses (LPNs/LVNs), aides/assistants, therapists, technologists/technicians, and community-based workers.^38,39^

### Measures and statistical approach

First, for each year of the survey, we calculated the percentage of women in each education and occupation group. We created a ratio of the average annual wage and salary income (heretofore referred to as “wage”) of employed women in each health care education and occupation group relative to the average annual wages of employed men in each education and occupation group within each year. Annual wages were inflated to 2020 dollars using the Consumer Price Index. We descriptively examined the trend over time of women's representation by education and occupation group and compared this to the ratio of women-to-men average annual wages. In addition, a descriptive table of wages for men and women by occupation and education (2003, 2012, and 2021) can be found in Appendix Table 1.

Second, using a pooled sample, we used a generalized linear model to estimate wages using a gamma distribution with a log link. To estimate the wage gap by gender, our main independent variable was gender. In our models, we controlled for the percentage of women in the workforce in the occupation in which the individual is working. We also included control variables of age, age-squared, underrepresented minority (URM) status (ie, non-Hispanic Black, Pacific Islander, or American Indian/Alaska Native individual as well as any Hispanic individual), immigrant status (US born vs naturalized/non-US citizen), marital status (married vs never married/separated/divorced/widowed), presence of any children (<18 years old) in the household, part-time status (<35 hours per week), urbanicity (living in metro area or not), living location in 1 of 9 US Census regions, and year indicators. We report predicted margins at the mean for wages for women vs men by highest education and occupational category.

## Results

### Descriptive analysis of women's representation and the gender wage gap across educational levels in the health care sector

**Figure 1.**
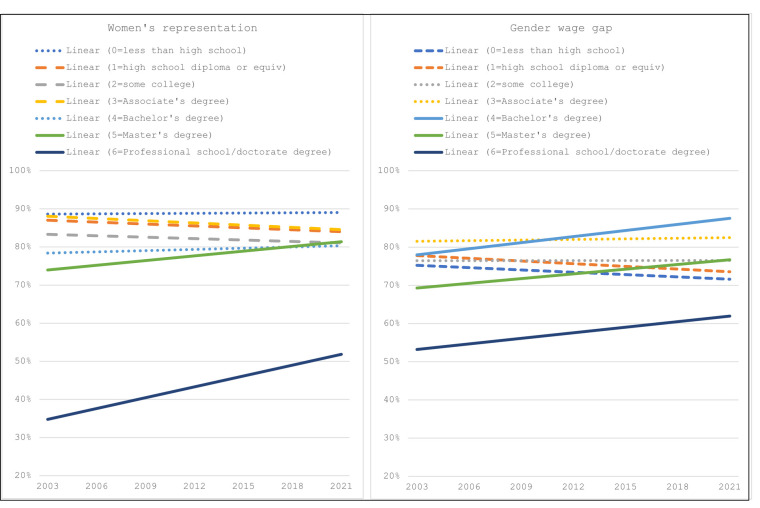
Women's representation and the gender wage gap by educational level. *n* = 190 733. Source: Current Population Survey (2003–2021). Dashed lines indicate a downward trend; dotted lines indicate a stagnant trend; solid lines indicate an increasing trend. Weights were used calculating women’s representation and the gender wage gap. In the figure, the “linear” label indicates that the figure includes linear trendlines, rather than yearly averages.

The educational level with the highest representation of women was less than a high school degree, which remained steady at just under 90% between 2003 and 2021 (Figure 1). Women's representation increased 8% at the master's degree level (from 76% in 2003 to 82% in 2021) and 42% at the professional/doctorate level (from 35% in 2003 to 50% in 2021). Women's representation among workers with a high school degree, some college, and an associate’s degree declined slightly (−3%, −5%, and −3% decreases in women's representation, respectively). Representation of women among those with a bachelor's degree remained steady (0% change) at approximately 80% since 2003.

Comparatively, in 2021, the gender wage gap was the lowest among workers with a bachelor's degree (88%), followed by those with an associate’s degree (82%), some college (77%), master's degree (77%), high school degree (72%), less than high school (71%), and professional school/doctorate degree (61%) (Figure 1). The gender wage gap narrowed between 2003 and 2021 among workers with a bachelor's (14% change), master's (11% change), and professional/doctoral degree (15% change), but the gender wage gap widened among those with lower levels of education, including those with a high school degree or less (−5% change) or a high school degree (−6% change).

### Descriptive analysis of women's representation and the gender wage gap across occupational groups in the health care sector

**Figure 2.**
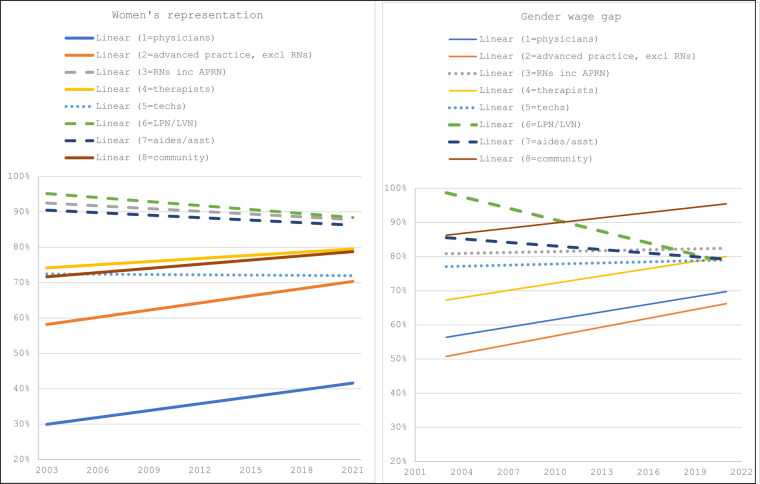
Women's representation and the gender wage gap by occupational group. *n* = 177 412. Source: Current Population Survey (2003–2021). Dashed lines indicate a downward trend; dotted lines indicate a stagnant trend; solid lines indicate an increasing trend. Weights were used calculating women’s representation and the gender wage gap. In the figure, the “linear” label indicates that the figure includes linear trendlines, rather than yearly averages. Abbreviations: APRN, advanced practice registered nurse; asst, assistants; excl, excluding; inc, including; LPN, licensed practical nurse; LVN, licensed vocational nurse; RN, registered nurse.

The occupational categories with the highest representation are the nursing occupations, including LPNs (89%), RNs (88%), and aides/assistants (86%), but all of these occupations have had a decrease in the representation of women since 2003 (−5% change) (Figure 2). The representation of women among technician occupational category remained steady since 2003 at approximately 72%. Representation of women between 2003 and 2021 increased among physicians (26% change), advanced practitioners (not including RNs; 15% change), therapists (7% change), and community-based workers (7% change).

In comparison, the gender wage gap was the narrowest among community-based workers (96%) and RNs (82%), with therapists, LPNs, aides/assistants, and technicians clustered together at approximately 80% in 2021 (Figure 2). The gender wage gap was the widest for physicians (70%) and advanced practitioners (68%). The gender wage gap improved between 2003 and 2021 among community-based workers (30% change), therapists (43% change), physicians (41% change), advanced practitioners (36% change), and technicians (9% change). The gender wage gap widened among aides/assistants (−11% change) and RNs (−5% change) over the study period.

### Regression of the gender wage gap across education and occupational categories

**Figure 3.**
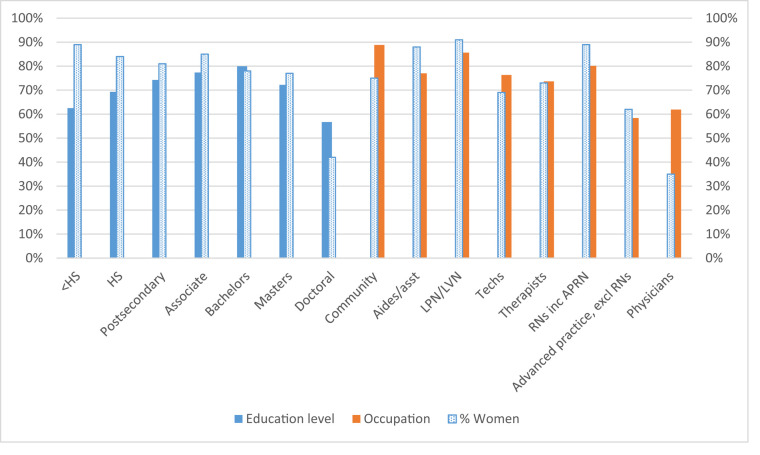
Women's wages relative to men's wages (adjusted). *n* = 177 412 (occupation); *n* = 190 733 (educational level). Source: Current Population Survey (2003–2021). Predicted women’s wages relative to men’s wages were calculated while controlling for the percentage of women in the occupation in which an individual works, age, age-squared, underrepresented minority (URM) status, being an immigrant (naturalized or non-US citizen), being married, having a child in the household, working part time, living in a metro area, living in 1 of 9 US Census regions, and year indicators. Abbreviations: APRN, advanced practice registered nurse; asst, assistants; excl, excluding; HS, high school; inc, including; LPN, licensed practical nurse; LVN, licensed vocational nurse; RN, registered nurse.

To test the relationship between annual wages and gender, we used a generalized linear model to estimate wages using a gamma distribution with a log link. To estimate the wage gap by gender, our main independent variable was gender. Our regression findings (Figure 3) mirrored the descriptive findings presented above; after controlling for several individual-level observable factors, we found that the gender wage gap was the largest for those with the lowest and highest levels compared with middle levels of education, with those with professional/doctoral-level degrees having the widest gap (59%), followed by those with less than a high school degree (61%). Those with a bachelor's degree and an associate’s degree both had the lowest gender wage gap, at 81%. In looking at occupation, the smallest wage gap was among community-based workers (86%), followed by technicians (78%), therapists (78%), RNs (78%), and aides/assistants (78%). Physicians (60%), LPNs (55%), and advanced practitioners (64%) had the largest wage gaps. In addition to showing women's wages as a percentage of men's wages (adjusted) in Figure 3, we also include the percentage of women for each educational and occupational group to show the relationship between women's representation and the gender wage gap.

### Supplemental analyses

We tested whether the trends held when the sample was restricted to full-time workers only (Appendix Figure 1); approximately 20% of our sample work part-time, with the percentage of part-time workers dropping slightly over time (from 22% in 2003 to 18% in 2021). The findings for full-time workers were consistent with our findings for all workers in the sample. In Appendix Figures 2 and 3, we show that the gap in absolute wages for men and women is wider when limiting the sample to full-time workers, across educational levels and occupation. Finally, in Appendix Figures 4 and 5, when controlling for hours as a continuous variable and limiting our sample to married workers only, we found that the gender wage gap was wider for workers across all educational levels and occupations among married workers compared with the full sample of workers.

## Discussion

We found that, over the last decade, women's representation dramatically increased over the last 2 decades in health care occupations that require high levels of education, including physicians, advanced practitioners, and therapists. For example, the percentage of women among physicians increased from 30% in 2003 to over 40% in 2021 in our sample (41% change), and the percentage of women among advanced practitioners increased from below 60% to over 70% over the last 2 decades (36% change). On the other hand, women's representation dropped slightly—and men's representation increased—in some health care occupations over time, including RNs, LPNs/LVNs, and aides/assistants. For example, in 2003, approximately 93% of RNs were nurses, but this dropped to approximately 88% in 2021. In general, occupations where women had lower representation 2 decades ago, like physicians, had strong growth in women's representation over this time period, while occupations that were heavily women-dominated, like nursing, saw men's representation increase during the same time period. This tradeoff in men’s vs women’s representation by occupation and education may be otherwise masked and appear to be stagnant in aggregate analyses of the health care industry.

The gender wage gap decreased over the last 2 decades in many health care occupations—a trend that occurred across the United States^40^—and notably among those where women's representation increased substantially. For example, among advanced practitioners, the gender wage gap decreased from women's earnings being approximately 50% of men's earnings to approximately 65% of men's earnings. We found a similar pattern among women and men physicians over the last 2 decades, although the gender wage gap among physicians remains much higher than the general population.^40^ In general, women with higher levels of education (professional degrees, master's degrees, or bachelor's degrees) made substantial gains in terms of wage equity relative to men in the health care sector.

However, for women with lower levels of education, the gender wage gap story is more complicated. The gender wage gap for RNs, technicians, LPNs/LVNs, and aides/assistants is, in general, smaller as compared with occupations that require higher levels of education. For example, women RNs made approximately 82% of men RNs, as compared with women physicians, who made approximately 70% of men physicians. However, in many lower-education, heavily women-dominated health care occupations, the gender wage gap widened over the last 20 years. For example, among aides/assistants, women earned approximately 85% of men in 2003, but this dropped to below 80% in 2021. Similarly, the gender wage gap for LPNs/LVNs and RNs remained basically stagnant or widened slightly over the last 2 decades. In these lower-wage jobs with high women representation, the gender wage gap is similar to what is seen nationally,^40^ but unlike national trends towards closing of the pay gap, it is widening or stagnant in these occupations over time.

To summarize, we found that women's representation in health care occupations increased to a greater degree in high-education health care occupations (eg, physicians, advanced practitioners), and the gender wage gap also improved substantially in many of these occupations during this time period. However, as described above, the gender wage gap remains larger in high-education occupations as compared with lower education health care occupations. In a few health care occupations, primarily within nursing, men's representation increased slightly over the last 2 decades, and women's representation dropped slightly. Further, in some of the lower education health care occupations, the gender wage gap remained stagnant or widened over the last 2 decades.

Past research on the glass escalator has shown that men in women-dominated occupations have higher wages and are more likely to be promoted compared with their women peers,^26,27^ and we argue that our findings support the glass escalator theory, particularly among lower wage occupations. Our findings suggest that, as more men enter heavily women-dominated health care occupations, like aide/assistant, LPN, and RN occupations, the gender wage gap is either stagnant over time or worsens. While the CPS does not allow us to examine employment characteristics at a granular level, the worsening gender wage gap is likely because men are being offered higher wages than their women counterparts, they are advancing into supervisory positions, and they are concentrated in specialties that have higher compensation.

It should be noted that the gender wage gap may be a function of both gender representation and education. There has been an improvement in the gender wage gap among those with a college degree and higher, while there has not been a closing of the gender wage gap among those with less than a college degree. Consequently, it is important to note that the gender wage gap may be operating differently for professional women as compared with women with less than a college degree.^2,22,41^ There are additional alternative explanations for the trends that we observe other than the glass escalator. For instance, as the gender wage gap goes up, women may decide to leave an occupation (reverse causality) or relatively high-earning women may be more likely to leave in search of better paying jobs than low-earning women (adverse selection). Marital status has also been associated with gender wage gaps. For example, a recent study of women physicians married to men physicians found that these women worked fewer hours than women who were not married to physicians.^42^ These trends are likely related to total family income and whether the health worker is the primary or secondary earner in their families.^43^ Being the secondary earner of the family gives someone more flexibility to exit the labor market or to choose reduced hours and/or wages.^44^ In the higher education/pay end, women health workers (women physicians, for example) are more likely to have high-education/pay spouses, and this assortive mating plus the social norm for women to take on more family responsibilities together may explain why so many women physicians work fewer hours or do not seek high pay as aggressively.^45^ Testing these explanations is beyond the scope of our paper but is an important area for future research in gender inequity across the health professions.

### Limitations

Our study has a few limitations. First, we looked only at the association between gender composition of the workforce and the gender wage gap, and we did not measure this relationship directly. Second, due to the complexity of our analysis and small sample size among health care workers, we did not break out the gender wage gap by race-ethnicity over time. As demonstrated in the literature review, there is significant variation in the gender wage gap by race-ethnicity, with women of color experiencing substantially greater disadvantages. Third, we do not have details on the roles and experience that may confer higher wages, such as supervisory roles, specialties, overall work experience, or tenure. Further, while the US Census is working towards expanding questions on gender identity, the ASEC currently only collects binary self-reported gender (male or women) and thus masking other potential sources of pay inequities.^46,47^

### Policy implications

#### Organizational policies

Our findings have significant implications for organizational policy and practices. Health care organizations need to be diligent in their evaluation of equity-based pay structures, which may include providing more pay transparency and taking a more activist role in making sure that women-typed credentials and skills are valued at comparable rates to credentials and skills that are not gendered or associated with men.^48-50^ Standardizing starting wages, evaluating and standardizing wage increases, and evaluating the rewards for different types of credentialing—and whether gender bias is associated with credentialing—are ways to address the chronic devaluation of women's work, especially women of color.^51^ There is also significant evidence that promoting women into leadership positions reduces the gender wage gap. A recent study in Britain found that the wage gap is eradicated when more than 60% of workplace managers are women, and that the association between the share of women managers and the gender wage gap is more pronounced when workplace managers set pay at the workplace and where employees are paid for performance.^52^

#### Federal and state policies

Finally, despite Medicare reimbursement rates being set at a national level, studies have found that women receive lower reimbursement for services than men, even after controlling for experience, productivity, and intensity. The differences have been attributed to differences in practice and billing approaches.^53,54^ Further, Medicare and Medicaid reimbursement policies and practices may promote gender wage inequality across health care occupations, with the highest rates and rewards being concentrated around services that are more male-dominated (eg, surgery, procedures involving technology), while services that require the most hands-on care and are most associated with femaleness are reimbursed with the lowest rates (eg, direct care work, long-term-care work). The relative pay of physician specialties in the United States is heavily influenced by the political power of medical boards, which exert a huge influence on the prices of medical procedures, and which may devalue procedures that are performed in more women-dominated specialties. Committees that set reimbursement rates, including within the Centers for Medicare and Medicaid Services (CMS), should consider gender and racial-ethnic equity within the health care workforce as critical goals when designing and realigning reimbursement rates, as well as ensure the committee is reflective of the diversity of the population; redistributing Medicare and Medicaid funds to promote greater gender and racial-ethnic wage equity can and should be important priorities of the CMS.^55^ Redistributing greater funding towards preventive care and population-based care may also impact the gender wage gap in health care, as many occupations that are involved in these types of care have high levels of women's representation.

## Conclusion

Health care is a women-dominated industry and should be a leader in promoting gender equity in wages. Given the high level of turnover experienced by women health care workers relative to men, accentuated during the pandemic,^39^ identifying tools to recruit and retain women, such as gender equity in payment is one important approach. Health care organizations and government agencies should strive to implement policies and practices that continue to improve the gender wage gap across all educational levels and occupations.

## Supplementary Material

qxad090_Supplementary_Data
