# Supplementary material for: The gender wage gap among health care workers across educational and occupational groups
Source: Health Aff Sch. 2023 Dec 27;2(1):qxad090. doi: 10.1093/haschl/qxad090 (PMC10986226; doi:10.1093/haschl/qxad090)
Supplement: qxad090_Supplementary_Data [file qxad090_Supplementary_Data.zip › Wage gaps_Appendix.docx]

Appendix Table 1. Mean wages by gender, occupation, and education

|  | **2003** | | **2012** | | **2021** | |
| --- | --- | --- | --- | --- | --- | --- |
|  | **Men** | **Women** | **Men** | **Women** | **Men** | **Women** |
|  | Mean | Mean | Mean | Mean | Mean | Mean |
|  | (SE of mean) | (SE of mean) | (SE of mean) | (SE of mean) | (SE of mean) | (SE of mean) |
|  | N | N | N | N | N | N |
| Physicians | $252,437 | $119,675 | $196,218 | $131,080 | $245,039 | $164,099 |
|  | $295 | $289 | $265 | $164 | $257 | $202 |
|  | 400 | 175 | 355 | 202 | 286 | 187 |
| Advanced practice, excl RNs | $129,802 | $69,722 | $125,219 | $72,700 | $109,349 | $81,976 |
|  | $235 | $99 | $233 | $88 | $127 | $78 |
|  | 343 | 514 | 314 | 651 | 267 | 588 |
| RNs inc APRN | $74,306 | $60,217 | $72,524 | $64,582 | $88,667 | $69,201 |
|  | $81 | $26 | $83 | $25 | $72 | $31 |
|  | 124 | 1,609 | 159 | 1,736 | 231 | 1,515 |
| Therapists | $53,922 | $32,943 | $63,103 | $35,398 | $56,380 | $51,501 |
|  | $128 | $50 | $379 | $42 | $106 | $158 |
|  | 59 | 186 | 70 | 298 | 58 | 235 |
| Technicians | $66,851 | $43,196 | $53,774 | $41,367 | $64,524 | $45,993 |
|  | $157 | $37 | $107 | $21 | $84 | $32 |
|  | 258 | 881 | 296 | 896 | 323 | 942 |
| LPN/LVN | $39,584 | $39,649 | $48,634 | $38,740 | $57,502 | $46,925 |
|  | $82 | $27 | $120 | $27 | $174 | $142 |
|  | 19 | 392 | 27 | 329 | 21 | 225 |
| Aides and assistants | $27,108 | $26,704 | $28,081 | $23,724 | $35,574 | $27,862 |
|  | $42 | $15 | $33 | $12 | $51 | $16 |
|  | 211 | 2,213 | 294 | 2,542 | 278 | 1,921 |
| Community-based workers | $57,326 | $43,652 | $48,985 | $43,144 | $49,138 | $50,564 |
|  | $91 | $23 | $56 | $25 | $47 | $38 |
|  | 340 | 848 | 242 | 785 | 185 | 764 |
| Less than high school | $51,181 | $20,894 | $30,602 | $18,057 | $54,094 | $26,688 |
|  | $527 | $21 | $118 | $21 | $392 | $66 |
|  | 41 | 353 | 30 | 315 | 14 | 205 |
| High school diploma or equiv | $41,767 | $29,001 | $32,966 | $26,898 | $38,444 | $29,070 |
|  | $105 | $13 | $47 | $13 | $53 | $26 |
|  | 190 | 1,417 | 180 | 1,309 | 154 | 963 |
| Some college | $46,435 | $34,099 | $43,670 | $30,326 | $40,630 | $29,303 |
|  | $124 | $18 | $162 | $24 | $45 | $16 |
|  | 218 | 1,234 | 215 | 1,199 | 203 | 852 |
| Associate's degree | $60,218 | $46,184 | $64,029 | $43,405 | $57,336 | $45,216 |
|  | $145 | $23 | $212 | $17 | $49 | $31 |
|  | 165 | 1,401 | 236 | 1,632 | 222 | 1,276 |
| Bachelor's degree | $79,825 | $56,756 | $65,012 | $56,760 | $71,470 | $61,186 |
|  | $100 | $37 | $71 | $26 | $63 | $33 |
|  | 458 | 1,772 | 458 | 2,046 | 468 | 1,860 |
| Master's degree | $103,854 | $68,039 | $92,505 | $64,486 | $94,542 | $68,410 |
|  | $198 | $61 | $140 | $59 | $160 | $33 |
|  | 263 | 732 | 255 | 934 | 245 | 1,167 |
| Professional school/doctorate | $206,931 | $97,735 | $168,513 | $98,796 | $186,320 | $122,311 |
|  | $226 | $175 | $200 | $92 | $167 | $117 |
|  | 636 | 328 | 578 | 474 | 531 | 527 |

Source: Current Population Survey

Appendix Figure 1. Women’s wages relative to men’s wages (adjusted) among FULLTIME workers only

Source: Current Population Survey (2003-2021), n=147,424 (education level), n=135,448 (occupation)

Notes: Predicted women’s wage relative to men’s wages were calculated while controlling for age, age squared, under-represented minority (URM) status, being an immigrant (naturalized or non-US citizen), being married, having a child in the household, working part time, living in a metro area, living in one of 9 Census regions, and year indicators. Weights were used when estimating the model.

Appendix Figure 2. Comparison of gender wage gap in adjusted wages between fulltime workers and full sample, by education level

Source: Current Population Survey (2003-2021), n=147,424

Notes: Predicted women’s wage relative to men’s wages were calculated while controlling for age, age squared, under-represented minority (URM) status, being an immigrant (naturalized or non-US citizen), being married, having a child in the household, working part time, living in a metro area, living in one of 9 Census regions, and year indicators. Weights were used when estimating the model.

Appendix Figure 3. Comparison of gender wage gap in adjusted wages between fulltime workers and full sample, by occupational category

Source: Current Population Survey (2003-2021), n=135,448

Notes: Predicted women’s wage relative to men’s wages were calculated while controlling for age, age squared, under-represented minority (URM) status, being an immigrant (naturalized or non-US citizen), being married, having a child in the household, working part time, living in a metro area, living in one of 9 Census regions, and year indicators. Weights were used when estimating the model.

Appendix 4. Women’s wages relative to men’s wages (adjusted) with HOURS WORKED PER WEEK INCLUDED AS A CONTINUOUS VARIABLE

Source: Current Population Survey (2003-2021), n=190,716 (education level), n=177,396 (occupation)

Notes: Predicted women’s wage relative to men’s wages were calculated while controlling for age, age squared, under-represented minority (URM) status, being an immigrant (naturalized or non-US citizen), being married, having a child in the household, hours per week, living in a metro area, living in one of 9 Census regions, and year indicators. Weights were used when estimating the model.

Appendix 5. Women’s wages relative to men’s wages (adjusted) among MARRIED WORKERS ONLY

Source: Current Population Survey (2003-2021), n=114,986 (education level), n=106,071 (occupation)

Notes: Predicted women’s wage relative to men’s wages were calculated while controlling for age, age squared, under-represented minority (URM) status, being an immigrant (naturalized or non-US citizen), being married, having a child in the household, working part time, living in a metro area, living in one of 9 Census regions, and year indicators. Weights were used when estimating the model.
